# Verbal and Non-Verbal Aggression in a Swiss University Emergency Room: A Descriptive Study

**DOI:** 10.3390/ijerph15071423

**Published:** 2018-07-06

**Authors:** Dominic Kaeser, Rebekka Guerra, Osnat Keidar, Urs Lanz, Michael Moses, Christian Kobel, Aristomenis K. Exadaktylos, Meret E. Ricklin

**Affiliations:** 1 Department of Emergency Medicine, Inselspital, University Hospital Bern, Freiburgstrasse, 3010 Bern, Switzerland; dominic.kaeser@gmail.com (D.K.); rebekkav@hotmail.com (R.G.); osnat.keidar@insel.ch (O.K.); drmikemoses@gmail.com (M.M.); aristomenis.exadaktylos@insel.ch (A.K.E.); 2 Security Service, Inselspital, University Hospital Bern, Freiburgstrasse, 3010 Bern, Switzerland; urs.lanz@insel.ch; 3 Legal service, Inselspital, University Hospital Bern, Freiburgstrasse, 3010 Bern, Switzerland; christian.kobel@insel.ch

**Keywords:** aggression, emergency department, workplace violence, migrants

## Abstract

Workplace violence (WPV) by patients and visitors is a hazard in many emergency departments (ED), with serious consequences for both staff and patients. Patients with a migratory background seem to be prone to being involved in WPV. We therefore reviewed all reports of ED staff who experienced WPV over a 4-year period (2013–2016). We analyzed data on the reasons for the incident, the time of day, the manner of violence, the consequences, and the migratory background of the aggressor. In total, 83 cases of WPV were reported over a four-year period. The average age of the violent person was 33.1 years; in 35 cases (42.0%), aggressors were younger than 30 years old, 53 (63.8%) were male, 49 (59%) were of Swiss nationality, and 35–40% had a migratory background. The odds ratio of people originating from a low- to middle-income country versus those originating from a high-income country was 1.8. Furthermore, 45.8% of the patients arrived by ambulance (*n* = 38) and 19 patients (22.9%) were self-presenting. Most cases (92.8%) involved verbal aggression, but in more than half of the cases, physical assault (56.6%) was also reported. In addition, 43 (51.8%) of the events occurred during the night. Results also showed that 42 (50.6%) of patients who were involved in WPV were under the influence of alcohol and 29 (34.9%) suffered from psychiatric disorders. Security personnel and police were involved in 53 (63.9%) and 47 (56.6%) cases, respectively. Twenty patients (24.1%) were sedated and 16 (19.3%) were restrained. In 18 cases (21.7%), the psychiatrist ordered compulsory hospitalization in a psychiatric institution. Taken together, WPV is a relatively common event in our ED and persons with a migratory background are involved more often relative to their frequency of ED visits.

## 1. Introduction

Workplace violence (WPV) by patients and visitors is a reality in many emergency departments (ED) all over the world [1,2,3,4,5,6,7,8,9]. Previous studies have shown that in North America and Great Britain, 74% of doctors were victims of verbal abuse (VA) and 28% were victims of physical abuse (PA) over a one year period. Furthermore, 81% of nurses experienced VA and 26% experienced PA over a one month period [10,11,12]. Depending on the setting, rates of VA may be up to 100% within the last twelve months. However, published data about WPV in the ED setting reports very different crime rates depending on the setting and location [13,14,15,16,17,18,19].

WPV is not only a hazard to the ED staff, but also to other patients as it can disturb the departmental workflow and impact patient safety [20]. In addition, WPV can lead to personal consequences, such as stress, increased rates of missed workdays, burnout, job dissatisfaction, high consumption of alcohol or drugs, relationship breakdown, and post-traumatic stress disorder [11,13,21].

Various risk factors have been reported to contribute to WPV. These include patient-related risk factors, such as social, economic and cultural factors, male gender, alcohol intoxication, substance abuse, mental disorder, and language barrier that leads to frustration [20,22,23,24]. These factors are often found in migrants, especially those who originate from low- and middle-income countries [25]. With these reasons and the recent increase of patients with migratory backgrounds seeking ED assistance, as well as previous reports indicating that migrants are prone to be at higher risk for aggressive behavior [26], the aim of this study was to define the incidence of and characterize cases of WPV that were reported at the ED of a Swiss university hospital. Our data indicates that the investigated ED had a relatively low level of WPV with an increased odds-ratio of people originating from low- to middle-income countries to be involved in aggressive behavior.

## 2. Materials and Methods

This single centre retrospective study that was done in a Level one university hospital emergency department in Bern, the capital of Switzerland, used the report folder for “threat and aggression” where all reports were filed by ED staff who experienced and reported WPV. The study included all cases of WPV where the patient was 16 years or older.

After identifying the cases from January 2013 to December 2016 and cross-referencing the information with our clinical information system, we generated an anonymised data spreadsheet. The cases were analysed by patient criteria, such as age, gender, nationality, migratory background, main diagnosis, and reason for hospital admission. Furthermore, we analysed the incidence, the categorisation of PA, VA, threat, sexual harassment, stalking, damage to property, or theft, and the time of the aggression according to shift time (07:00–15:00; 15:00–23:00; 23:00–07:00). Necessary interventions were grouped by the involvement of police and/or security personnel, psychiatrist consultation, use of sedative medication, or restraint of the patient. Possible consequences included letters of complaint, expulsion from the hospital, refusal of future admissions to the hospital area, pressing charges against the patient, or compulsory hospitalisation in a psychiatric institution ordered by the psychiatrist. The cases were analysed by year in order to identify trends and developments.

According to the international organization for migration (IOM), a migrant is any person who is moving or has moved across an international border or within a State away from his/her habitual place of residence [27]. However, we used a different categorization. We calculated the odds and odds ratio for people of low- to middle-income countries versus high-income countries with a gross national income per capita of above $12,236 for 2018 using Microsoft Excel [28].

The study was performed according to Swiss law and consent of the cantonal ethic commission was given (KEK No. 390/15).

## 3. Results

The study center was an emergency department of a Swiss Level one university hospital with a catchment area of about 2 million people, which treats about 45,000 patients per year [29]. In the time period of the study, 159,388 patients were seen in total, with 13.7% of patients originating from low- to middle-income countries. A total of 84 cases of WPV were reported from January 2013 to December 2016. No cases were excluded. From January to December 2013, there were 4.5 cases of WPV per 10,000 patients who were treated in the ED, in 2014 there were 6.3 cases, in 2015 there were 4.9 cases, and in 2016 there were 4.3 cases per 10,000 patients.

The mean age of the aggressors was 33.1 years. In 53 cases (63.9%), the aggressor was male. Thirty-five (42.0%)—23 males and 12 females—were between 16 and 30 years of age (Table 1). The gender of the perpetrator was not recorded in seven cases, nor was it possible to determine it properly. The same was true for the age of the perpetrator in six cases.

Over the study period, WPV incidences were between 17 and 25 per year. And of these perpetrators, we observed a twofold increase between 2013 and 2016—from 7 to 14 cases. Most patients possessed a Swiss passport (49; 59.2%) or passports from other high-income countries (23; 31.9%). The percentage of patients with a migratory background with origins in a low- to middle-income country who were involved in WPV was 22%. In comparison to this, 22% of foreigners in total live in the Bern area [30]. Compared to high-income countries, patients from low- and middle-income countries had an elevated incidence of aggression with an odds ratio of 1.81.

Most patients were admitted to the hospital by ambulance (45.9%) or were self-presenting (22.9%), and nine (10.8%) were brought to the department by the police.

The most common main diagnosis on admission was intoxication with alcohol (*n* = 42) or other substances (*n* = 11). Next were mental disorders (34.9%) without additional intoxication. Most cases of mental disorders were suicidal ideation (26.1% of psychiatric patients), followed by schizophrenia (17.4% of psychiatric patients). Twelve (19.0%) aggressors were admitted to the ED due to injuries. Eighteen (21.7%) patients were treated for internal medical problems other than intoxication.

Most cases of WPV involved VA (92.8%); in half of the cases, PA (56.6%) was involved. In 24.1% of cases, there were threats against the ED staff, and five cases (6.0%) of property damage and two cases (2.4% of patients) of sexual harassment were also reported. In five cases, relatives were involved in WPV and in four cases, a weapon was present.

In total, 15 (18.1%) cases of WPV occurred between 07:00 and 15:00, 24 (28.9%) between 15:00 and 23:00, and 43 (51.8%) between 23:00 and 07:00 (Table 2).

Assistance by internal security personnel was necessary in 63.9% of cases and in 56.6% of cases, the police were involved. Psychiatrist consultation was requested in 34.9% of patients who were involved in WPV, in 24.1% sedative medication was requested, and in 19.3% of cases, fixation was deemed necessary.

More than half of the patients (53%) who were involved in WPV received a letter of complaint from the hospital. Six patients (7.2%) were told to leave the hospital area and 3 patients (3.6%) were banned from returning to the hospital area. In 21.7% of cases, a psychiatrist ordered compulsory hospitalisation in a psychiatric institution. This figure increased from zero in 2013 to ten in 2015 and dropped to five in 2016. In 9.6% of cases, charges were pressed against the patient, however it is unknown how often this led to a conviction. The charges included four PA and four with PA in combination with sexual harassment, damage of property, and threat. Nineteen (29%) patients who were involved in PA (55.8%) received a letter of complaint, charges were pressed against seven (20.6%), and in seven cases (9.5%), the aggression had no consequences.

## 4. Discussion

The aim of this study was to characterize the reported WPV over a 4-year period from January 2013 to December 2016 in the ED of a Swiss Level one university hospital and to describe the associated factors, necessary interventions, and the consequences of these incidents.

Even though the personnel anecdotally reported an increase in cases of WPV, actual reported cases did not increase over the last few years. However, numbers of reported cases were very low. It is not certain if the number of reported cases is a true image of the situation. It may be that awareness of WPV is increasing, but that the staff remain reluctant to accept the importance of reporting it. Most studies estimate that 50% to 75% of cases are unreported [31]. The principle barriers to reporting are fear of retaliation, absence of physical injury, fear of inconvenience from reporting WPV, or the potential effect on customer satisfaction; ED staff often think that it is an inherent part of the job [32].

It is easy to imagine the impact on staff and patients—especially if the large numbers of unreported cases are considered—to be similar to that in previous studies [13,19]. In the present study, we believe that there may be a high degree of underreporting, since only the most serious cases were reported—those with a major consequence to or significant impact on the affected person. This is shown by the fact that in 2014, only 4 of 25 cases were reported where there was no PA or threat involved and in nearly 60% of all cases, intervention by the police was necessary. Those cases are very severe. On one hand, the fact that these make up 60% of the reported cases indicates that only the top of the iceberg is being reported. On the other hand, it indicates that in the ED of this study, we have a selection bias of cases as small institutions do not need to accept to treat the agitated, intoxicated, and partially persons with a migratory background as this University ED does.

In other studies, stalking or theft were mentioned as further categories of WPV, but in our cases, no such incidences were identified [13].

Comparable to other studies, most patients were young and male [13]. The number of patients with a migratory background from low- to middle-income countries in WPV was stable, with 35 to 40%—what is about double of the foreign population living in the Bern region, which was 22% in 2016 [33]. In the same period, the frequency of asylum seekers attending the ED increased by 45% from 465 to 653 per year [29]. Unfortunately, in most of the studies that were published about WPV, no information about the migratory background of the aggressors can be found. However, a study by Knutzen et al. showed that the rate of the use of restraint in the emergency department was significantly higher in patients with an immigrant background, especially in younger age groups, and a Swedish study reported violence as one of the problems that nurses experienced by working with migrants in emergency care [30,34]. In our study, the data did not allow us to draw conclusions about the motive or reason of the patients for the violence. However, other studies show that factors like the potential language barrier, cultural differences, or differences in the health care system might lead to frustration and therefore to aggression [34,35,36]. To meet the need of this increasing population who are visiting the ED, our ED regularly trains the health care professionals on intercultural competence in health care by the Red Cross. Other methods for de-escalation are to address the psychological and emotional distress and the unmet needs of the people involved. Furthermore, skilled communication with no confrontation, trust-building, and negotiation are suggested to be good ways to manage critical situations and to avoid harm [37].

The risk factors for WPV that have been found in this study were the same as in prior studies [13]. These were mainly male gender, alcohol intoxication, substance abuse, and mental disorders. Apart from male gender, these are all factors that lead to scenarios where the patient may not be able to rationally interpret the situation and might feel threatened or fear for loss of dignity. The severity of the cases shown in this study and the fact that many cases involved more than one category of aggression show that patients who might not be capable of coping with these unusual circumstances use every possible action to defend themselves from what they see as a threat [20,22,23]. An additional risk factor in our population was admission to hospital by ambulance or police.

Because of the lack of available information, it was not possible to identify other risk factors, such as waiting time, pain, and surroundings, or to show the impact of WPV for ED staff, as for data protection reasons, we were not able to obtain the interviews of either the patients who were involved in the WPV or the staff. Another limitation of this single center study is the low caseload of WPV, despite the high amount of people who were seen. To shed more light on the situation, a better reporting system might be needed, and to describe the situation in Switzerland, a multicenter study might be helpful that includes all large EDs.

As reported before, nearly all cases of WPV included VA [13]. VA might be the starting point for the escalation of the situation and therefore, could be a point for the prevention of PA. A study from Fernandes et al. from 2002 showed that there was an initial decrease in violent events 3 months after violence reduction workshops. However, there was a slight increase by 6 months after the workshop—what was assumed to be a positive effect of the training that was extinguished over time—but this could possibly be regained with refresher courses [38]. For threat and sexual harassment, nearly half of the cases involved PA, and security personal and police were almost always involved. Most cases happened between 23:00–07:00, when fewer staff were present and the number of patients with alcohol intoxication was highest. Only 8 of 47 cases of PA happened between 07:00–15:00. Most cases where restraint was necessary occurred between 15:00–23:00 or 23:00–07:00. Hyland et al. analysed the cases of aggressive behaviour of the patients over a 12-month period in an Australian ED and found a similar trend, with 76.5% of cases of WPV from 17:00–08:00 [39]. These findings show that security personnel ought to be rapidly available at all times and especially during night shifts.

## 5. Conclusions

In this single center study, we described the 4-year incidence of WPV in a Swiss university hospital. Patients with a migratory background originating from low- or middle-income countries, compared to high-income countries, had an elevated prevalence of aggression with an odds ratio of 1.8 to show aggressive behavior. Other factors that could be related to aggression were male gender, alcohol intoxication, substance abuse, and mental disorders.

As only one tool of WPV reporting was available and the nurses mainly reported the events, we have to assume that substantial numbers of the cases were not reported. We recommend the introduction of a reporting platform where all ED staff, including security personnel, can rapidly and simply report WPV. Furthermore, it will be necessary to improve staff awareness of the importance of reporting cases of WPV. The number of cases and the time period in this study were relatively small. Future studies should include more cases in order to confirm and extend the findings and to assess the effect of preventive measures taken, such as staff training and access to intercultural translators. Furthermore, we suggest qualitative studies in parallel to understand in detail the motivation of aggression and sequelae for the staff.

## Figures and Tables

**Table 1:** Characteristics of the aggressor.

Characteristic	Total	16–20 AIY	21–30 AIY	31–40 AIY	41–50 AIY	51–60 AIY	>60 AIY
Male	50 (59.5)	6 (7.2)	18 (20.5)	11 (13.3)	5 (6.0)	3 (3.6)	7 (8.4)
Female	27 (32.1)	5 (6.0)	8 (8.4)	8 (9.6)	1 (1.2)	4 (4.8)	1 (1.2)
Unknown	7 (8.4)						
Nationality	Swiss	Western E	East E	African	Asian	Other/Unknown	
	50 (59.2)	9 (10.8)	8 (9.6)	3 (3.6)	4 (4.8)	10 (12)	
Country of Origin	L&M-IC	H-IC	Unknown				
	17	56	12				
Year	2013	2014	2015	2016	Total		
Total of WPV cases	17	25	22	20	84		
RR M	0.4	0.4	0.29	0.35	0.31		
Admission Self-admission Ambulance	Self-admission	Ambulance	Police	Psychiatrist	Physician	Other/Unknown	
	19 (22.9)	38 (45.9)	9 (10.8)	3 (3.6)	5 (6.0)	9 (10.8)	
Diagnosis	Psychiatric	Alcohol	Intox	Injury	Other	Unknown	
	29 (34.9)	42 (50.6)	11 (17.5)	12 (19.0)	18 (21.7)	6 (7.2)	

AIY: Age in years, N (%). Multiple diagnoses may be given. E. = Europe; L&M-IC: low- and middle-income country; H-IC: high-income country. Intoxications were not included under psychiatric diseases; alcohol intoxication was not included under intoxication. 7 were of unknown gender. RR of M = relative risk of persons with a migratory background to be involved in WPV.

**Table 2:** Case characteristics of WPV.

Characteristic	N (%)	N (%)	N (%)	N (%)	N (%)	N (%)
Time of incidents	07:00–15:00	15:00–23:00	23:00–07:00			
	15 (18.1)	24 (28.9)	43 (51.8)			
Type of aggression	Verbal	Physical	Sexual	Threat	Damage	
	77 (92.8)	47 (56.6)	2 (2.4)	20 (24.1)	5 (6)	
Intervention	Security	Police	Psychiatrist	Sedatives	Fixation	
	53 (63.9)	47 (56.6)	29 (34.9)	20 (24.1)	16 (19.3)	
Consequences	None	Expulsion	Refusal of admission	Letter of complaint	äFU	Charges pressed
	8 (9.6)	6 (7.2)	3 (3.6)	44 (53.0)	18 (21.7)	8 (9.6)

äFU = compulsory hospitalization in a psychiatric institution; RR = relative risk of migrants to be involved in WPV.
